# Predictive value of hepatitis B serological indicators for mortality among cancer survivors and validation in a gastric cancer cohort

**DOI:** 10.1371/journal.pone.0286441

**Published:** 2023-12-27

**Authors:** Yangyu Zhang, Linlin Qu, Yuchen Pan, Yanhua Wu, Jing Jiang

**Affiliations:** 1 Department of Epidemiology and Biostatistics, School of Public Health, Jilin University, Changchun, China; 2 Department of Clinical Epidemiology, The First Hospital of Jilin University, Changchun, China; 3 Department of Laboratory Medicine, The First Hospital of Jilin University, Changchun, China; 4 Center of Infectious Diseases and Pathogen Biology, The First Hospital of Jilin University, Changchun, China; Dalhousie University, CANADA

## Abstract

Hepatitis B virus (HBV) infection has gradually been considered to associate with cancer development and progression. This study aimed to explore the associations of serological indicators of HBV infection with mortality risk among cancer survivors and further validated using a gastric cancer (GC) cohort from China, where HBV infection is endemic. National Center for Health Statistics’ National Health and Nutrition Examination Survey (NHANES) data were used in this study. Individuals with positive results of hepatitis B core antigen (anti-HBc) were considered to have current or past HBV infection. Serological indicators were positive only for hepatitis B surface antibodies (anti-HBs), indicating vaccine-induced immunity, whereas negativity for all serologic indicators was considered to indicate the absence of HBV infection and immunity to HBV. The GC cohort included patients from the First Hospital of Jilin University, China. The median follow-up time of the NHANES was 10 years; during the follow-up, 1505 deaths occurred. The results revealed that anti-HBs-positive cancer survivors had a 39% reduced risk of mortality (hazard ratio [HR] 0.61, 95% confidence interval [CI] 0.44–0.85). Men and individuals aged <65 years old with past exposure to HBV had higher mortality risk (HR 1.52, 95% CI 1.09–2.13; HR 2.07, 95% CI 1.13–3.83). In this GC cohort, individuals who were only anti-HBs-positive showed a reduced risk of mortality (HR 0.77, 95% CI 0.62–0.95). Thus, anti-HBs positivity was a significant factor of decreased mortality among cancer survivors. More rigorous surveillance is necessary for cancer survivors with anti-HBc positivity, particularly men, and younger individuals.

## Introduction

Hepatitis B virus (HBV) infection is a critical public health problem globally; nearly 30% of the global population demonstrates serological evidence of current or past HBV infection and that 257,000,000 individuals currently live with chronic HBV infection [1]. In recent years, aside from hepatocellular carcinoma (HCC), HBV infection has been reported to be associated with the development and progression of various extrahepatic tumors, such as lung cancer [2], lymphoma [3], esophageal cancer [4], colorectal cancer [5], and gastric cancer (GC) [6].

The loss of hepatitis B surface antigen (HBsAg) is considered a safe endpoint for the termination of acute or chronic HBV treatment, known as a functional cure [7]. However, even if HBsAg is cleared, a true cure may not be feasible because of the integration of the HBV DNA into the host genome and the persistence of covalently closed circular DNA (cccDNA) [8]. In cases with HBsAg clearance, the hepatitis B core antigen (anti-HBc) is often the only detectable marker of a past HBV infection. Traditionally, anti-HBc is regarded as the indicator for past or ongoing HBV infection and is used as a blood screening test in HBV low-to-medium epidemic regions [9, 10]. A relatively higher anti-HBc level was found to be associated with the presence of the cccDNA in transplant donors with occult HBV infection, and a cross-sectional study reported that a higher anti-HBc level was associated with occult HBV among HBsAg-negative individuals [11, 12]. Li et al. found that anti-HBc positivity is associated with a higher risk of early intrahepatic recurrence and poorer relapse-free survival of patients with HBV-related HCC following curative resection [13]. These findings indicated that the anti-HBc presence may demonstrate the presence of non-eradicated HBV.

Cancer is expected to become the leading cause of death and the most important barrier to extending life expectancy worldwide in the 21st century. Globally, the incidence and mortality of cancer are growing rapidly [14]. Cancer survivors are a rapidly growing group, which changes the population structure, promotes the popularization of early diagnosis and screening and improved treatment and survival [15]. Approximately 16,900,000 adults have cancer, and 1,600,000 new cases are diagnosed annually in the USA [16]. Thus, the identification of novel prognostic markers is essential for prognosis evaluation, early intervention, and personalized therapy in cancer survivors. In cancer survivors, damage to the host immune system because of using chemotherapeutic or immunosuppressive agents increases the risk of HBV activation [17]. The European Association for The Study of The Liver practice guidelines suggest that among patients receiving chemotherapy or immunosuppressive therapy, individuals who were HBsAg-negative and anti-HBc-positive should receive anti-HBV prophylaxis if they have a high risk of HBV reactivation [18]. However, procedures for medical supervision after HBsAg clearance have not been well established, and evidence on the effect of past HBV exposure on mortality in cancer survivors remains scarce.

Hepatitis B surface antibody (anti-HBs) is a commonly used indicator for hepatitis B and immune status screening, and its role also deserves attention. Previous studies have revealed the need for full anti-HBs response after HBV vaccination, which may be influenced by inflammation or health status [19]. Thus, the anti-HBs response has the potential for use as a significant predictor of mortality among cancer survivors.

This study aimed to use data from the US nationally representative sample of the National Center for Health Statistics’ National Health and Nutrition Examination Survey (NHANES) to evaluate the associations of serological indicators of hepatitis B with risk of mortality among cancer survivors. Moreover, the effect of the serological indicators of hepatitis B on mortality was further validated using a GC cohort from China, where HBV infection is endemic.

## Methods

### Data source

This study used NHANES data from 1999 to 2018, including 10 surveys over 2 years [20]. To identify a nationally representative sample of noninstitutionalized civilian population, the NHANES used a stratified, multistage, probability cluster sampling design. Interviews and physical examinations were conducted. The interview covers demographics, dietary, socioeconomics, and health. Medical, physiological, and laboratory tests, etc., were also performed.

The NHANES survey was approved by the institutional review board of the US Centers for Disease Control and Prevention (CDC). Informed consent was obtained from all sampled persons (for those aged <18 years, assent with proxy consent). All data were de-identified and made publicly available. More detailed descriptions of the NHANES survey are available on the CDC website [20].

### Cancer status

Data on cancer diagnosis and cancer type were derived from self-reported health status information in the medical conditions section. Participants were surveyed, “Have you ever been told by a doctor or other health professionals that you had cancer or a malignancy of any kind?” Participants who responded “yes” were defined as cancer survivors and were asked, “What kind of cancer was it?” and “How old were you when this cancer was first diagnosed?” [21]. Only cancer survivors were enrolled in this study. Participants who had missing diagnosis time or were <18 years old at diagnosis were excluded.

### Cancer site

Cancer site subgroups were divided according to the anatomical site or system affected according to National Cancer Institute [22]: skin nonmelanoma, melanoma, skin unknown cancers, breast, gynecological (ovary, cervix, and uterus), genitourinary (kidney, bladder, prostate, and testis), and digestive/gastrointestinal (esophagus, stomach, liver, gallbladder, pancreas, colon, and rectum). The remaining cancer types with fewer cases were classified as “others.” Participants with cancer of unknown kinds were excluded.

### Variables and definitions

Immunoassays were used to test the following HBV serological indicators: anti-HBc, HBsAg, and anti-HBs. The HBsAg test was performed only in individuals who were positive for anti-HBc. Participants who tested negative for anti-HBc did not undergo HBsAg testing and were considered negative for HBsAg. Participants who were positive for anti-HBc were recognized as having current or past HBV infection. Serological indicators that were positive only for anti-HBs indicated vaccine-induced immunity, whereas negative results for all serologic markers (anti-HBc and anti-HBs) indicated noninfection and nonimmunity to HBV. A positive anti-HBs test was defined as anti-HBs titer >10 IU/L from 1999 to 2006 and >12 IU/L from 2007 to 2018. Participants without data for serologic markers of HBV infection and participants with positive hepatitis C antibody were excluded. The variables and definitions of self-reported sociodemographic characteristics are described in S1 File.

### Mortality

The NHANES Linked Mortality File was used to obtain the mortality status [23]. Individuals from NHANES 1999–2014 were linked through probabilistic matching with multiple identifiers to the US National Death Index and were followed up to the date of death or December 31, 2015 [24]. If no matched death records were obtained, individuals were considered alive, and censored at the end of the follow-up. Participants without mortality outcome information were excluded.

### Patients of GC selection

This retrospective observational cohort study enrolled patients newly diagnosed with GC who were admitted to the First Hospital of Jilin University between August 2008 and January 2019. The inclusion criteria were as follows: (1) histopathologically confirmed primary GC, (2) radical gastrectomy and no chemotherapy or radiotherapy before surgery, and (3) available serological indicator of HBV infection, including HBsAg, anti-Hbs, and anti-HBc. The exclusion criteria were as follows: (1) distant metastasis, (2) lost to follow-up at the first appointment, (3) death within 30 days after the operation, and (4) coinfection with the hepatitis C virus.

This study was approved by the Ethics Committee of the First Hospital of Jilin University (Changchun, China, 2013–005). The need for informed consent was waived owing to minimal risk, and patient data were collected and analyzed anonymously.

### Data collection and follow-up of patients with GC

Data on the clinical characteristics of the included patients were retrieved from an electronic medical record system of the hospital. These included sex, age, tumor size, histological grade, T stage, N stage, neural invasion, vascular invasion, and postoperative chemotherapy. Follow-up was conducted regularly at 3 months, 6 months, and 1 year after the radical gastrectomy and then annually until death or loss to follow-up.

### Serologic assay for HBV markers

Serological indicators of HBV infection were obtained through the medical record system. Serum levels of the three HBV serological indicators were determined using chemiluminescent immunoassay (CLIA) by I4000 (Abbott, USA) following standard procedures. A positive anti-HBs test result was defined as anti-HBs titer >10 IU/L.

### Statistical analysis

Owing to the complex sampling design, sampling weights were used in the analysis of NHANES data. Sociodemographic, physical examination, and lifestyle factors were compared by HBV infection status (all negative, exposed to hepatitis B, and only anti-HBs-positive). The Chi-square test was used to compare categorical variables. These characteristics are summarized with unweighted sample sizes, weighted percentages, and *P* values. Multivariable Cox proportional hazards regression models were applied to estimate hazard ratios (HR) and 95% confidence interval (CI) for the associations of HBV infection status with overall mortality. In addition, subgroup analyses were conducted on the cancer site. Sensitivity analyses were performed by excluding individuals diagnosed with liver cancer or diagnosed with cancers within 1 year. In the CG cohort, the same statistical method was used, without using weighted data. Missing values of the GC cohort were interpolated using the R software “mice” package (with five imputed datasets and random forest method). The IBM SPSS Statistics version 26.0 (IBM Corp., Armonk, NY, USA) and Stata 13.0 (College Station, TX, USA) were used to conduct all analyses. Statistical significance was considered a two-sided *P* value <0.05.

## Results

### Characteristics of cancer survivors in the NHANES

**Fig 1:**
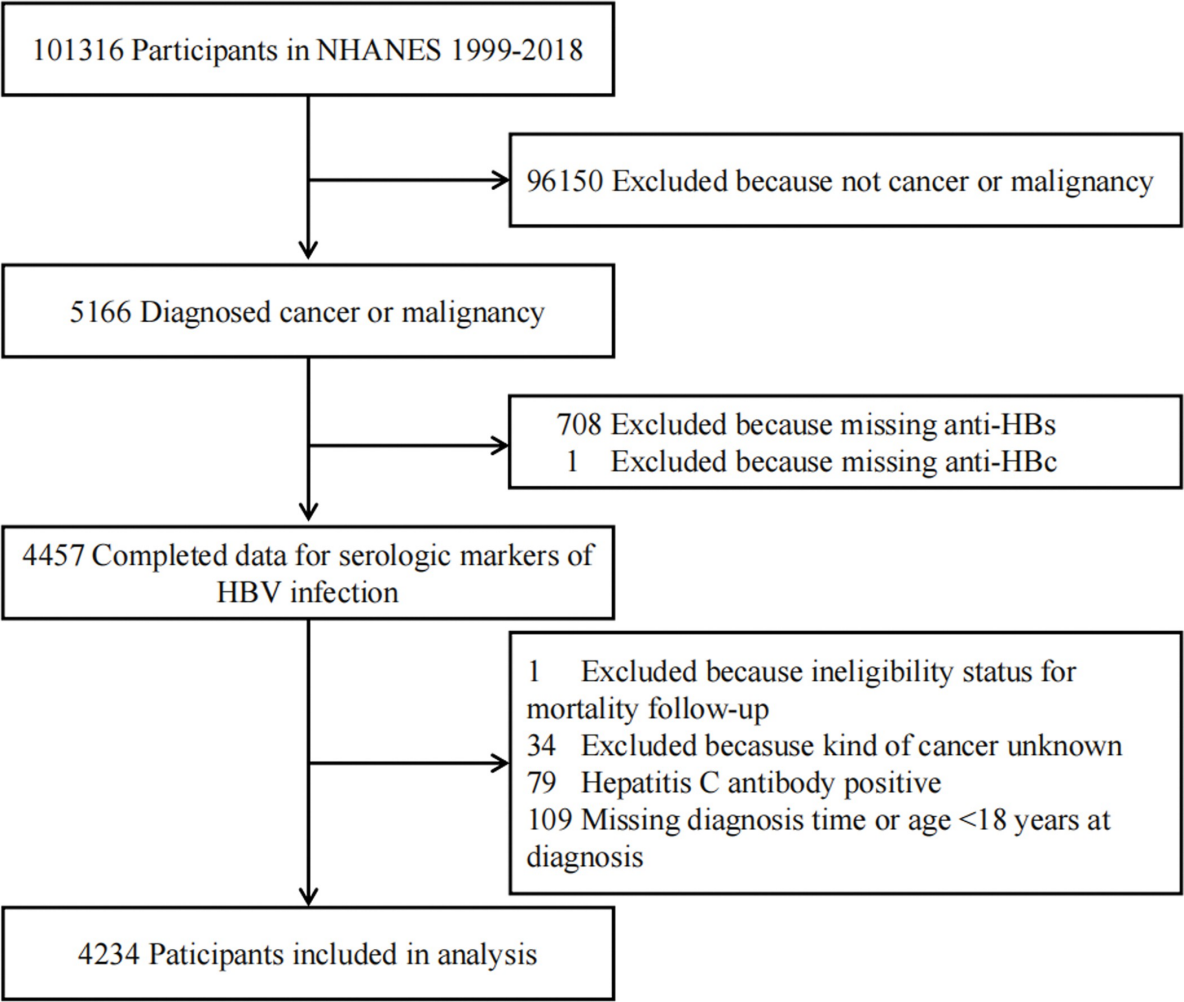
Flowchart of participants in the National Health and Nutrition Examination Survey (NHANES) from 1999 to 2018.

A total of 101,316 individuals participated in 10 consecutive NHANES 2-year cycles (1999–2018). Of those individuals, 5,166 were diagnosed with cancer or malignancy. Finally, 4,234 participants were involved in the study (Fig 1). The distribution of hepatitis B infection status of the participants with different demographic and health-related characteristics is shown in Table 1. Participants who were more likely to be exposed to HBV had the following characteristics: being male, being of non-Hispanic Black, or other race, born outside the USA, never married, being a current smoker, and not having health insurance.

**Table 1:** Distribution of the characteristics of cancer survivors by HBV infection status.

	All negative	Exposed to hepatitis B	Only anti-HBs-positive	*P* value
**Overall**	3608(84.6)	261(4.2)	365(11.2)	−
**Sex**				<0.01
Male	1731(86.3)	152(5.6)	128(8.2)	
Female	1877(83.3)	109(3.3)	237(13.5)	
**Age group**				<0.01
<65 years	1265(78.7)	96(4.4)	228(16.9)	
≥65 years	2343(90.1)	165(4.1)	137(5.8)	
**Race**				<0.01
Mexican American	262(88.3)	13(2.9)	26(8.8)	
Other Hispanic	166(73.7)	20(8.3)	23(18.0)	
Non-Hispanic White	2661(72.7)	115(3.3)	227(10.7)	
Non-Hispanic Black	400(72.7)	88(15.4)	57(11.9)	
Other races	119(68.7)	25(9.8)	34(21.5)	
**Place of birth**				<0.01
USA	3199(85.1)	198(3.7)	315(11.2)	
Non-USA	407(77.3)	63(11.6)	50(11.1)	
**BMI (kg/m** ^**2**^ **)**				0.53
≤24.9	995(84.3)	83(4.6)	112(11.2)	
25–29.9	1275(85.4)	82(4.4)	106(10.2)	
≥30	1250(83.8)	83(3.7)	141(12.5)	
**Education**				<0.01
≤High school	1688(88.6)	128(4.7)	104(6.7)	
≥ College or AA degree	1917(82.3)	132(4.0)	261(13.8)	
**Marital status**				<0.01
Married/living with partner	2196(83.9)	145(4.0)	239(12.1)	
Widowed/divorced/separated	1220(87.3)	91(4.2)	93(8.5)	
Never married	169(77.1)	24(8.2)	32(14.8)	
**Poverty index**				0.06
<1.3	757(84.0)	75(6.9)	57(9.1)	
1.3–3.5	1367(85.7)	101(4.0)	126(10.3)	
>3.5	1484(84.0)	85(3.7)	182(12.3)	
**Smoking**				0.02
Never smoker	1585(82.6)	119(4.3)	200(13.2)	
Former smoker	1512(87.5)	98(3.8)	109(8.7)	
Current smoker	511(83.1)	44(5.4)	56(11.5)	
**Alcohol**				0.04
Non-drinker	470(87.6)	31(3.9)	40(8.5)	
Former drinker	946(87.0)	76(4.7)	73(8.3)	
Current drinker	2015(83.5)	133(4.0)	227(12.5)	
Unknown	177(80.3)	21(6.7)	25(13.0)	
**Cardiovascular disease**				0.004
Yes	925(88.3)	71(4.9)	55(6.8)	
No	2683(83.6)	190(4.1)	310(12.3)	
**Diabetes**				0.002
Yes	686(89.2)	64(4.5)	54(6.3)	
No	2922(83.7)	197(4.2)	311(12.1)	
**Hypertension**				0.006
Yes	2047(86.4)	159(4.5)	171(9.1)	
No	1561(82.7)	102(4.0)	194(13.3)	
**Health insurance**				<0.01
Yes	3398(85.2)	239(4.0)	336(10.8)	
No	198(74.9)	21(7.0)	29(18.1)	
**Diagnosis time**				0.37
< 5 year	1258(83.3)	109(5.0)	131(11.7)	
≥ 5 year	2350(85.2)	152(3.9)	234(10.9)	

Distributions of characteristics were described as unweighted counts with weighted percentages.

### HBV infection status and mortality outcomes of NHANES

**Fig 2:**
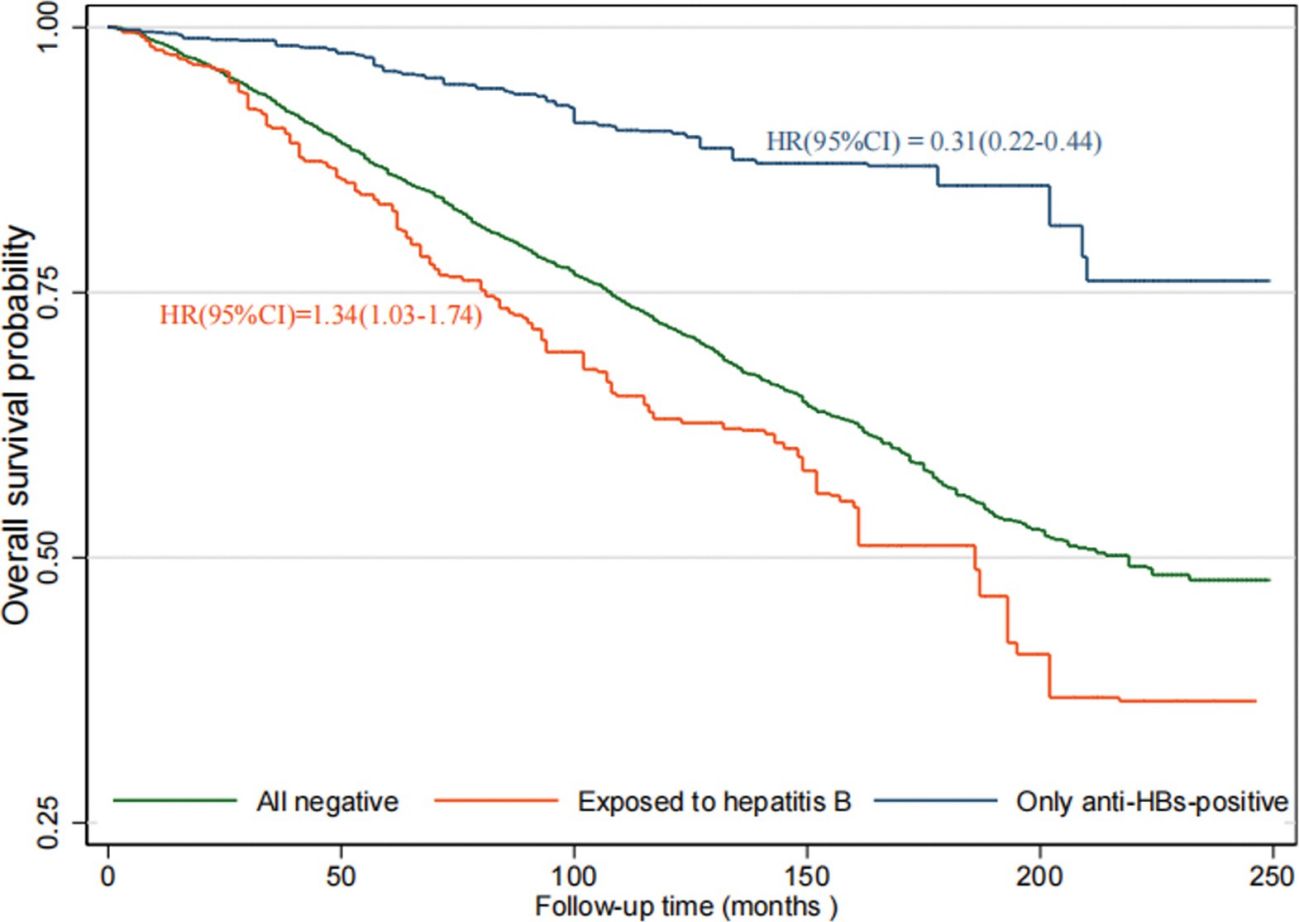
Kaplan–Meier estimates of overall survival with different HBV states in NHANES 1999–2018 (Reference: All negative). HR, hazard ratios; CI, confidence interval.

The median follow-up time was 10 years. During the follow-up, 1505 deaths occurred. Compared with individuals with all-negative HBV serologic markers, those who were exposed to hepatitis B had increased all-cause mortality risks, whereas individuals who were only anti-HBs-positive had decreased all-cause mortality risks (Fig 2). After adjusting for sex, age group, race, place of birth, BMI, education level, marital status, poverty index, smoking, alcohol, cardiovascular disease, diabetes, hypertension, health insurance, and time of diagnosis, the HR and 95% CI for all-cause mortality among individuals who were exposed to hepatitis B and individuals who were only anti-HBs-positive compared with those individuals with all-negative HBV serologic markers were 1.24(0.95–1.62) and 0.61(0.44–0.85), respectively (Table 2).

**Table 2:** Association of HBV infection status with all-cause mortality among US cancer survivors.

HBV status	Model 1	Model 2	Model 3
	HR (95% CI)	HR (95% CI)	HR (95% CI)
**All negative**	Ref	Ref	Ref
**Exposed to hepatitis B**	1.45(1.13–1.86)	1.34(1.03–1.75)	1.24(0.95–1.62)
**Only anti-HBs-positive**	0.54(0.39–0.74)	0.57(0.41–0.79)	0.61(0.44–0.85)

HR (95% CI), hazard ratios and 95% confidence interval. Model 1: Adjusted for age group and sex; Model 2: Further adjusted for BMI, smoking, and alcohol based on model 1; Model 3: Further adjusted for race, place of birth, education level, marital status, poverty index, cardiovascular disease, diabetes, hypertension, health insurance, and time of diagnosis based on model 2.

### Stratified analysis of cancer survivors in the NHANES

**Fig 3:**
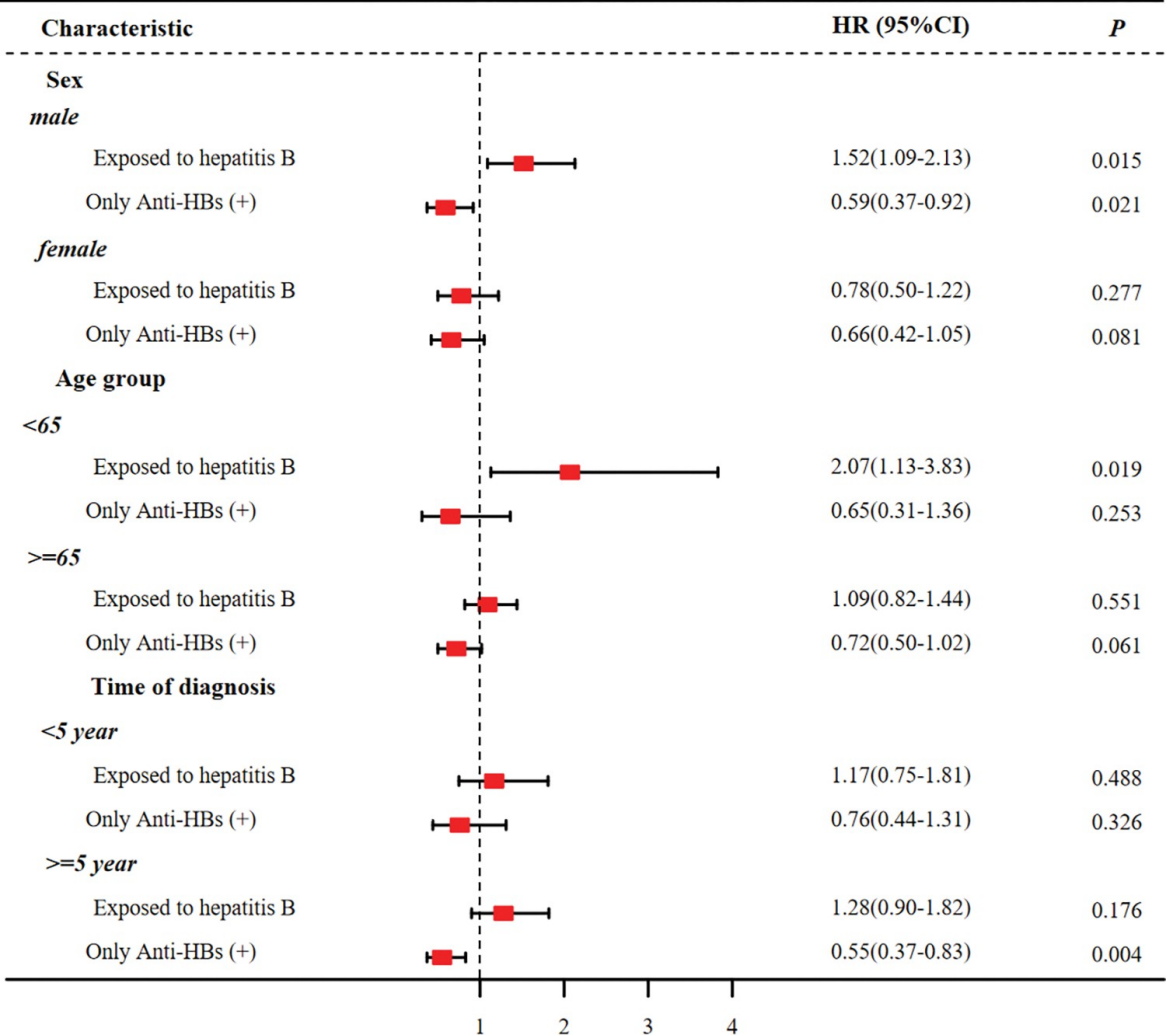
Stratified analysis of the association of HBV infection with the risk of all-cause mortality among US cancer survivors. HR (95% CI), hazard ratios and 95% confidence interval. All adjusted for age group, sex, race, place of birth, BMI, education level, marital status, poverty index, smoking, alcohol, cardiovascular disease, diabetes, hypertension, health insurance, and time since cancer diagnosis.

Fig 3 shows the stratified analysis by sex, age group, and time of diagnosis. Male participants who had current or past HBV infection have a 52% elevated risk of mortality (HR 1.52, 95% CI 1.09–2.13), and participants aged <65 years had a twofold risk of mortality (HR 2.07, 95% CI 1.13–3.83). Male participants who were only anti-HBs-positive (HR 0.59, 95% CI 0.37–0.92) and time of cancer diagnosis ≥5 years (HR 0.55, 95% CI 0.37–0.83) had a reduced risk of mortality. Differences in mortality by hepatitis B infection status were not found among women, group aged ≥65 years, and those with cancer diagnosed <5 years.

### Subgroup analysis of the cancer site of cancer survivors in the NHANES

In the cancer site-specific analyses, after adjustments were made for the potential confounding factors, an only anti-HBs-positive status was associated with a decreased risk of mortality among gynecological survivors (HR 0.19, 95% CI 0.04–0.84). Moreover, in the subgroup of digestive/gastrointestinal cancer with only 356 cases, participants who were exposed to HBV exhibited increased mortality risks (HR 2.15, 95% CI 1.23–3.76) (Table 3). Even after excluding participants with liver cancer, this risk could still be observed (HR 2.15, 95% CI 1.22–3.79). Therefore, based on our GC cohort, which included a larger number of patients with GC, we further validated the correlation between hepatitis B infection status and mortality among cancer survivors.

**Table 3:** Association of HBV infection status with all-cause mortality of a specific site among cancer survivors (Reference: All negative).

	Cases	All negative	Exposed to hepatitis B		Only anti-HBs-positive	
		N	N	HR (95% CI)	N	HR (95% CI)
Skin non-melanoma	673	595	28	0.95(0.41–2.23)	50	0.36(0.13–1.01)
Melanoma	258	234	6	1.37(0.53–3.56)	18	1.81(0.46–7.09)
Skin unknown type	329	281	20	0.93(0.54–1.96)	28	0.54(0.20–1.44)
Breast	639	550	27	0.96(0.46–2.00)	62	0.85(0.45–1.61)
Gynecological	547	445	31	0.32(0.07–1.52)	71	0.19(0.04–0.84)
Genitourinary	866	731	75	1.36(0.78–2.35)	60	0.48(0.20–1.14)
Digestive/gastrointestinal	356	307	30	2.15(1.23–3.76)	19	0.77(0.30–2.00)
Other	566	465	44	1.48(0.77–2.84)	57	1.16(0.59–2.30)

HR (95%CI), hazard ratios and 95% confidence interval. All adjusted for age group, sex, race, place of birth, BMI, education level, marital status, poverty index, smoking, alcohol, cardiovascular disease, diabetes, hypertension, health insurance, and time since cancer diagnosis.

### Sensitivity analyses of cancer survivors in the NHANES

In the sensitivity analysis, after excluding individuals diagnosed with cancers within 1 year (N = 375), an only anti-HBs-positive status was also found to be associated with a decrease in mortality risk by 36% (HR 0.64, 95% CI 0.45–0.91). Meanwhile, the results remained similar in the sensitivity analyses after excluding 15 individuals with liver cancer (HR 0.62, 95% CI 0.44–0.86) (S1 Table). Sensitivity analyses were also performed in the subgroups of gynecological and digestive/gastrointestinal cancer, and the results were robust (S1 Table).

### Characteristics of the GC cohort

The GC cohort included 874 patients with GC. The demographic and clinical characteristics of the 874 patients are presented in S2 Table. The median (p25–p75) age of the enrolled patients was 61 (54–69) years, and most patients were male (75.5%). The median survival time of the enrolled patients was 49 months, and the 5-year survival rate was 45.7%. The correlation between different HBV infection states and clinicopathological features are presented in Table 4. Individuals who had HBV infection or were only anti-HBs-positive were older at diagnosis (*P* = 0.02). Moreover, HBV infection was significantly associated with larger tumors (*P* = 0.04).

**Table 4:** Relationships between HBV infection status and patient characteristics.

Characteristics	HBV infection status, N(%)			*P*
	All negative	Exposed to hepatitis B	Only Anti-HBs positive	
**Total**	375(42.9)	261(29.9)	238(27.2)	-
**Sex**				0.15
Male	271(41.1)	203(30.8)	186(28.2)	
Female	104(48.6)	58(27.1)	52(24.3)	
**Age group**				0.02
<65	251(46.5)	150(27.8)	139(25.7)	
≥65	124(37.1)	111(33.2)	99(29.6)	
**BMI(kg/m** ^**2**^ **)**				0.21
≤24.9	331(43.7)	139(26.1)	229(30.2)	
25–29.9	38(35.8)	38(35.8)	30(28.3)	
≥30	6(60.0)	2(20.0)	2(20.0)	
**Smoking**				0.47
Never smoker	225(42.2)	148(27.8)	160(30.0)	
Former smoker	0(0.0)	2(0.5)	2(0.5)	
Current smoker	150(44.5)	88(26.1)	99(29.4)	
**Alcohol**				0.82
Non-drinker	278(43.2)	276(27.3)	190(29.5)	
Former drinker	1(100.0)	0(0.0)	0(0.0)	
Current drinker	96(41.9)	62(27.1)	71(31.0)	
**T stage**				0.11
I	24(34.3)	20(28.6)	26(37.1)	
II	38(38.4)	26(26.3)	35(35.4)	
III	248(45.1)	170(30.9)	132(24.0)	
IV	65(41.9)	45(29.0)	45(29.0)	
**N stage**				0.24
N0	87(40.7)	56(26.2)	71(33.2)	
N1	71(40.1)	60(33.9)	46(26.0)	
N2	72(48.6)	41(27.7)	35(23.6)	
N3	145(43.3)	104(31.0)	86(25.7)	
**Tumor size**				0.04
<5cm	176(41.9)	114(27.1)	130(31.0)	
≥5 cm	199(43.8)	147(32.4)	108(23.8)	
**Differentiation**				0.49
Middle	97(41.3)	67(28.5)	71(30.2)	
Low	278(43.5)	194(30.4)	167(26.1)	
**Lymphovascular invasion**				0.52
No	89(40.6)	64(29.2)	66(30.1)	
Yes	286(43.7)	197(30.1)	172(26.3)	
**Neural invasion**				0.12
No	142(39.0)	113(31.0)	109(29.9)	
Yes	233(45.7)	148(29.0)	129(25.3)	

### HBV infection status and mortality outcomes of the GC cohort

**Fig 4:**
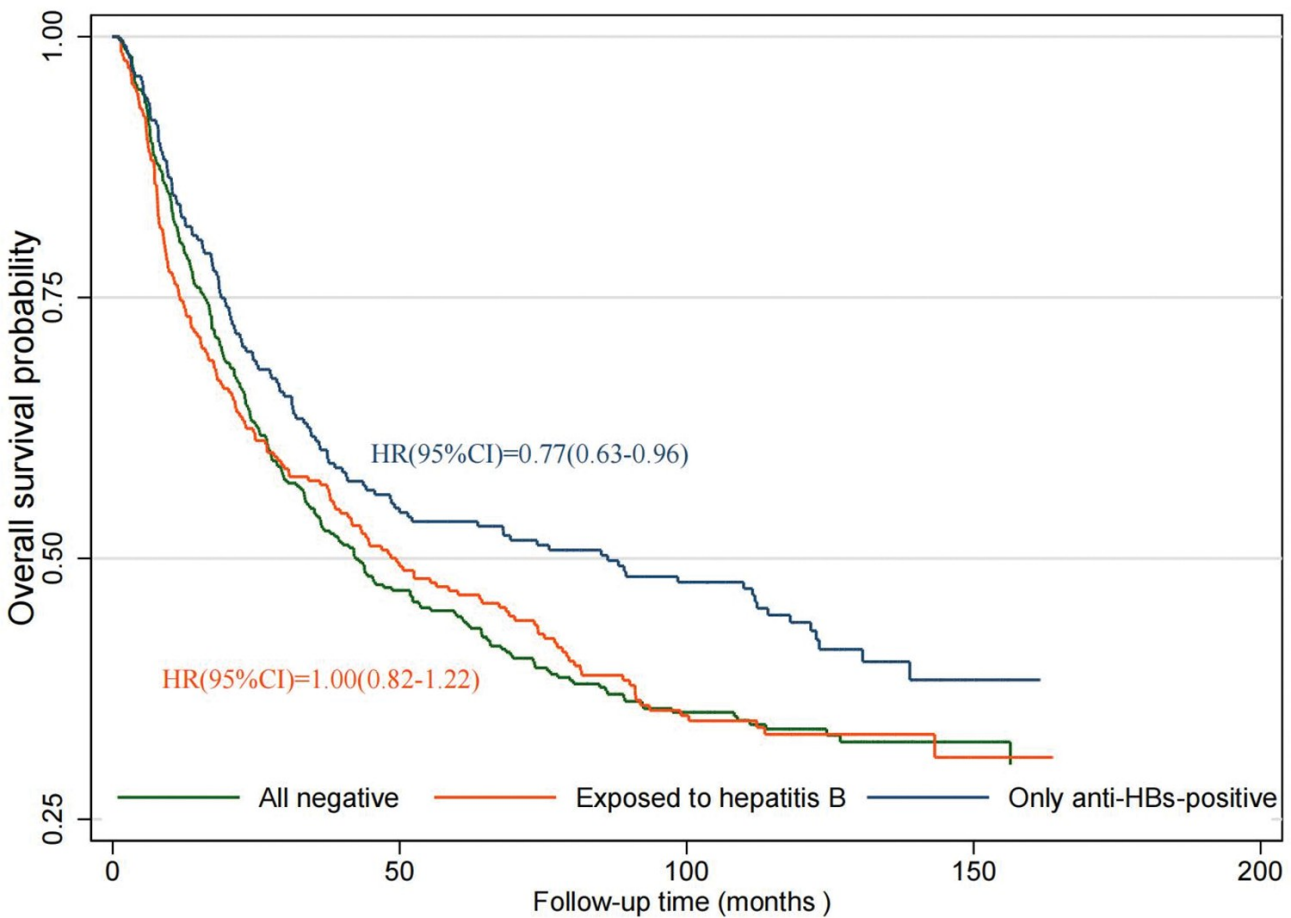
Kaplan–Meier estimates of overall survival with different HBV states in the gastric cancer cohort. HR, hazard ratios; CI, confidence interval.

During the follow-up period up to August 2022, 545 (62.4%) patients died, 194 (22.2%) were alive, and 135 (15.4%) were lost to follow-up. Individuals who were only anti-HBs-positive had a 23% decreased mortality risk (Fig 4). After adjusting for demographic and clinicopathological characteristics, HRs (95% CI) for mortality risk of individuals who were only anti-HBs-positive were 0.77 (0.62–0.95). Compared with individuals who were negative for all HBV serological indicators, those with HBV infection had insignificant increased mortality risk after adjusting for confounding factors (Table 5). In the stratified analysis, individuals aged <65 years (HR 0.69, 95% CI 0.51–0.93) and those without chemotherapy (HR 0.74, 95% CI 0.55–1.00) (S1 Fig) among individuals who were only anti-HBs-positive had decreased mortality risk.

**Table 5:** Association of HBV infection status with overall survival among patients with gastric cancer.

HBV status	Model 1	Model 2	Model 3
	HR (95% CI)	HR (95% CI)	HR (95% CI)
**All negative**	Ref	Ref	Ref
**Exposed to hepatitis B**	0.97(0.80–1.18)	0.794(0.80–1.87)	0.97(0.79–1.18)
**Only anti-HBs-positive**	0.75(0.61–0.93)	0.75(0.61–0.93)	0.77(0.62–0.95)

HR (95% CI), hazard ratios and 95% confidence interval. Model 1: Adjusted for age group, sex; Model 2: Further adjusted for BMI, smoking, and alcohol based on model 1; Model 3: Further adjusted for T stage, N stage, tumor size, differentiation, lymphovascular invasion, and neural invasion based on model 2.

## Discussion

In this analysis of a nationally representative cancer survivor cohort in the USA, 4.2% of the individuals had evidence of current or past HBV infection. To our knowledge, this is the first study to prospectively evaluate the HBV infection status with mortality risk in a nationally representative sample of cancer survivors. By using a survey with a median follow-up of 10 years, cancer survivors who were only anti-HBs-positive showed decreased all-cause mortality risks. Higher mortality rates were only found in men and individuals aged <65 years with past HBV exposure. In the cancer site-specific analyses, the analysis of the digestive/gastrointestinal carcinoma subgroup with a limited number of cases indicated that individuals with current or past hepatitis B infection had a higher mortality risk. Therefore, a cohort with 874 patients with GC was used in this study to further validate the correlation between hepatitis B infection status and mortality among cancer survivors. This GC cohort was from China, in which HBV infection is highly endemic and had the highest burden of HBV in the world. In this GC cohort, increased mortality was not found in individuals with past HBV exposure, whereas consistent with the finding of the NHANES, individuals who were only anti-HBs-positive still had significantly reduced mortality risk.

The association between HBV infection and prognosis of many cancer types has been previously described. HBV infection has been reported to be a poor prognostic factor in patients with nasopharyngeal carcinoma and breast cancer [25, 26]. However, Feng et al. found that patients with primary cervical cancer who had past HBV infection had better survival [27]. Most previous studies have recognized HBsAg as indicator of HBV infection. However, the overall prevalence of chronic HBV infection in the USA was approximately 0.3% in 2013–2018 [28], and the prevalence of HBsAg in the Chinese population of all ages was 7.8% [29]. In this study, only 10 (0.2%) and 27 (3.1%) patients were positive for HBsAg in the NHANES and GC cohorts, respectively. The lower prevalence rate of HBsAg may be attributed to the older age of the study participants, and the mean ages of the NHANES participants and GC cohort were 66 and 61 years, respectively. Chu et al. [30] explored the rates of HBsAg seroclearance in a long-term follow-up study and found that the cumulative probability of HBsAg seroclearance was 8.1% after the first 10 years, 24.9% after 20 years, and 44.7% after 25 years. Furthermore, studies conducted in China, Korea, and Japan have revealed that the age of spontaneous HBsAg seroclearance was approximately 50 years [31–33]. The presence of anti-HBc indicates recovery from an acute infection and is a meaningful indicator of past HBV exposure and infection [34]. An HBsAg-negative and anti-HBc-positive status can imply resolved infection; however, this may mean occult HBV infection in some individuals [35]. A study conducted in Italy, enrolling participants who underwent abdominal surgery from different regions and without liver disease, detected HBV DNA in the liver tissues, revealing occult HBV infection in 16% [36]. Hence, our study results focused more on anti-HBc, considering that the incidence of cancer is increasing in older individuals [37]. Despite this, the prognostic effect of active (HBsAg positive) on mortality stilled been examined in the two cohorts. In the NHANES, patients with active HBV showed a trend of worst survival; however, the difference between active HBV and past HBV infection did not reached a statistical difference (*P* = 0.732, S2A Fig). Similarly, no statistically significant difference was found in the mortality rates between these two subgroups in the GC cohort (*P* = 0.526, S2B Fig).

After adjusting for confounding factors, men and individuals aged <65 years with past HBV exposure and digestive/gastrointestinal carcinoma subgroup had higher mortality rates. However, the increased risk of mortality was not found among individuals with past HBV exposure in the GC cohort. The NHANES database allowed us to follow the individuals over an average >10 years. However, the average follow-up time in our GC cohort was only 5 years, which may partially explain the inconsistent findings. In addition, the digestive/gastrointestinal cancer subgroup only included seven types of cancer, with colon cancer being the most common and gastric cancer the second. Li et al. reported that higher titers of anti-HBc predict a poor prognosis in patients with colon cancer [38]. In our GC cohort, we also attempted to explore the effect of the titers of anti-HBc on patient survival. The results showed that individuals with higher titers of anti-HBc had a higher risk of death, but this did not reach statistical significance (S3 Fig).

How HBV infection affects cancer progression and the increase in mortality among cancer survivors with HBV infection remains to be elucidated. There may be some correlation with virus reactivation. Yeo et al. revealed that the risk factors of HBV reactivation include male sex and younger age [39], which are the characteristics that we study found to be associated with past HBV exposure and higher mortality risk. In addition, this could be related to HBV X-interacting protein [40], subsequent chronic inflammation [41], or certain types of immunological dysfunction [42]. Cui et al. also found higher expression levels of viral oncogenic HBV X protein in GC tissues, indicating that chronic HBV infection in gastric tissues may induce carcinogenesis through viral oncoprotein [6].

Cancer survivors who were only anti-HBs-positive were found to have a significantly decreased all-cause mortality risk in NHANES and GC cohorts. An only anti-HBs-positive status indicates being immune from past vaccination. Consistent with our results, previous studies have found that seroconversion and serum protection after active hepatitis B vaccination are significantly associated with reduced mortality in patients undergoing accidental dialysis [19], and HBV vaccination was also found to be a favorable predictor in patients with cervical cancer [43]. A meta-analysis that explored the role of anti-HBs in patients with resolved infection and hematologic malignancy revealed that the presence of anti-HBs is associated with reduced reactivation risk among all subgroups [44], whereas HBV infection reactivation may accelerate death in patients with severe disease [45]. The ability of anti-HBs to inhibit HBV infection reactivation may partially lead to reduced mortality in cancer survivors. Individuals who were only anti-HBs positive in our study tended to have better health behaviors. However, confounding factors have been adjusted in the multifactor model, and the protective effect of anti-HBs is stable, suggesting that these better health behaviors cannot fully explain the effect of anti-HBs on reducing mortality risk. Anti-HBs acquired by vaccination against HBV may be associated with cellular and humoral immunity, which might have some beneficial effects on cancer development and can be beneficial for long-term treatment. The stratified analysis of our study also showed that an only anti-HBs positive status had a significant predictive value for long-term outcomes (diagnosis time >5 years) in cancer survivors.

This study has some limitations that need to be considered. First, cancer diagnosis was based on self-report and may be influenced by recall bias. Second, data on tumor- and therapy-related characteristics such as the stage at diagnosis and treatment modality in the NHANES database were lacking. To minimize the potential effects of confounding factors, potential predictors such as age, race/ethnicity, sex, race, place of birth, BMI, education, marital status, poverty index, smoking, alcohol, disease history, and health insurance were adjusted. In addition, information on cancer stages, and treatment in the GC cohort were collected, which we believed have reduced the effect of this limitation on the results. Third, hepatitis B serological indicators were tested at baseline, and changes during the follow-up may not be reflected. Finally, further investigations on the biological mechanism underlying the association of HBV infection status with mortality among cancer survivors are needed.

An only anti-HBs-positive status was an independent predictor of decreased mortality among cancer survivors, which could be a meaningful indicator for monitoring related decisions. Past HBV exposure was associated with high mortality risk among men and individuals aged <65 years. This indicated that more rigorous monitoring is necessary for cancer survivors who were anti-HBc positive, particularly men, and younger individuals. More studies on the biological mechanism underlying the association between HBV serological indicators and mortality risk among cancer survivors are needed.

## Supporting information

S1 File Variables and definitions of self-reported sociodemographic characteristics. (DOC)

S2 File Data set. (XLS)

S3 File STROBE-checklist. (DOCX)

S1 Table Sensitivity analysis of the association of HBV infection with the risk of all-cause mortality among US cancer survivors. All adjusted for age group, sex, race, place of birth, BMI, education level, marital status, poverty index, smoking, alcohol, cardiovascular disease, diabetes, hypertension health insurance, and time since cancer diagnosis.
(DOC)

S2 Table Demographic and clinicopathologic characteristics of the enrolled patients with gastric cancer. (DOC)

S1 Fig Stratified analysis of the association of HBV infection with the risk of all-cause mortality in the gastric cohort. All adjusted for age group, sex, BMI, smoking, alcohol, T stage, N stage, tumor size, differentiation, lymphovascular invasion, neural invasion, chemotherapy, and time since cancer diagnosis.
(DOC)

S2 Fig Kaplan–Meier estimates of overall survival with different HBV infection states. A. NHANES; B. Gastric cancer cohort.
(DOC)

S3 Fig Kaplan–Meier estimates of overall survival with different titers of anti-HBc (cutoff: 8.42 S/CO). (DOC)
