# Supplementary material for: Predictive value of hepatitis B serological indicators for mortality among cancer survivors and validation in a gastric cancer cohort
Source: PLoS One. 2023 Dec 27;18(12):e0286441. doi: 10.1371/journal.pone.0286441 (PMC10752528; doi:10.1371/journal.pone.0286441)
Supplement: S1 File — (DOC) [file pone.0286441.s001.doc]

**Variables and definitions of self-reported sociodemographic characteristics**

Self-reported sociodemographic characteristics included gender (male, female), age (<65 years old, >=65 years old), race/ethnicity (Mexican American, other Hispanic, non-Hispanic White, non-Hispanic Black, other race), country of birth (born in the U.S. or Non-U.S.), educational attainment (<=High school, >=College or AA degree), marital status (Married/living with partner, Widowed/divorced/separated, Never married) and poverty index (<1.3, 1.3-3.5, >3.5). The health insurance was obtained from questionnaire data (Yes, No). Height and weight were acquired during the physical examination and body mass index (BMI; calculated as weight in kilograms divided by height in meters squared) was divided into three groups (<24.9, 25.0-29.9, >=30). Smoking status (Never, Former, Current) and alcohol use (Never, Former, Current, Unknown) were included in lifestyle factors. “Never smoker” was defined as individuals reported not smoked at least 100 cigarettes in their lifetime; “Current smoker” was defined as individuals reported smoke every day or some days at the time of the survey; Individuals smoked at least 100 cigarettes in their lifetime, but not current smokers were categorized as “Former smokers”. Individuals who had not consumed at least 12 alcoholic beverages in their lifetime were classified as “Non-drinker”; having drank at least 1 alcoholic beverage in the last 12 months were classified as “Current drinker”; consumption of at least 12 alcoholic beverages in their lifetime, but had not drunk any alcoholic beverages within the last 12 months were classified as “Former drinkers”. A history of cardiovascular disease (coronary heart disease, congestive heart failure, heart attack, angina, and stroke). Diabetes was self-reported by participants who had been previously diagnosed with diabetes or if they were taking prescribed medications for diabetes. Hypertension was defined as those participants who have received a prior diagnosis from a health professional or have an NHANES measured the blood pressure of ≥130 mm Hg systolic or ≥80 mm Hg diastolic.
