# Supplementary material for: Predictive value of hepatitis B serological indicators for mortality among cancer survivors and validation in a gastric cancer cohort
Source: PLoS One. 2023 Dec 27;18(12):e0286441. doi: 10.1371/journal.pone.0286441 (PMC10752528; doi:10.1371/journal.pone.0286441)
Supplement: S1 Table — All adjusted for age group, sex, race, place of birth, BMI, education level, marital status, poverty index, smoking, alcohol, cardiovascular disease, diabetes, hypertension health insurance, and time since cancer diagnosis. (DOC) [file pone.0286441.s004.doc]

**S1 Table. Sensitivity analysis of the association of HBV infection with the risk of all-cause mortality among US Cancer Survivors.**

| **HBV stutas** | **HR (95%CI) of All** | **HR (95%CI) of Gynecological** | **HR (95%CI) of Digestive/gastrointestinal** | |
| --- | --- | --- | --- | --- |
| **Excluding diagnosed< 1 year** | |  |  |  |
| All negative | Ref | Ref | Ref |  |
| Exposed to hepatitis B | 1.30(0.98-1.72) | 0.32(0.07-1.50) | 1.97(1.01-3.83) |  |
| Only Anti-HBs (+) | 0.64(0.45-0.91) | 0.22(0.06-0.90) | 0.84(0.32-2.18) |  |
| **Excluding liver cancer** | |  |  |  |
| All negative | Ref | - | Ref |  |
| Exposed to hepatitis B | 1.21(0.93-1.59) | - | 2.15(1.22-3.79) |  |
| Only Anti-HBs (+) | 0.62(0.44-0.86) | - | 0.99(0.40-2.47) |  |

All adjusted for age group, sex, race, place of birth, BMI, education level, marital status, poverty index, smoking, alcohol, cardiovascular disease, diabetes, hypertension health insurance and time since diagnosis of cancer
