# Supplementary material for: Predictive value of hepatitis B serological indicators for mortality among cancer survivors and validation in a gastric cancer cohort
Source: PLoS One. 2023 Dec 27;18(12):e0286441. doi: 10.1371/journal.pone.0286441 (PMC10752528; doi:10.1371/journal.pone.0286441)
Supplement: S2 Table — (DOC) [file pone.0286441.s005.doc]

**S2 Table.** **Demographic and clinicopathologic characteristics of the enrolled gastric cancer patients in the cohort.**

| **Characteristics** | **N** | **%** |
| --- | --- | --- |
| **Sex** |  |  |
| male | 660 | 75.5 |
| female | 214 | 24.5 |
| **Age group** |  |  |
| <60 | 387 | 44.3 |
| ≥60 | 487 | 55.7 |
| **BMI(kg/m^2^)** |  |  |
| <=24.9 | 758 | 86.7 |
| 25-29.9 | 106 | 12.1 |
| >=30 | 10 | 1.1 |
| **Smoking** |  |  |
| Never smoker | 533 | 61.0 |
| Former smoker | 337 | 38.6 |
| Current smoker | 4 | 0.5 |
| **Alcohol** |  |  |
| Non-drinker | 644 | 73.7 |
| Former drinker | 229 | 26.2 |
| Current drinker | 1 | 0.1 |
| **T stage** |  |  |
| I | 70 | 8 |
| II | 99 | 11.3 |
| III | 550 | 63 |
| IV | 155 | 17.7 |
| **N stage** |  |  |
| N0 | 214 | 24.5 |
| N1 | 177 | 20.3 |
| N2 | 148 | 16.9 |
| N3 | 335 | 38.3 |
| **Tumor size** |  |  |
| <5cm | 420 | 48.1 |
| ≥5 cm | 454 | 51.9 |
| **Differentiation** |  |  |
| Middle | 235 | 26.9 |
| Low | 639 | 73.1 |
| **Lymph vascular invasion** |  |  |
| No | 219 | 25.1 |
| Yes | 655 | 74.9 |
| **Neural invasion** |  |  |
| No | 364 | 41.6 |
| Yes | 510 | 58.4 |
| **Chemotherapy** |  |  |
| No | 430 | 49.2 |
| Yes | 444 | 50.8 |
