# Supplementary material for: Predictive value of hepatitis B serological indicators for mortality among cancer survivors and validation in a gastric cancer cohort
Source: PLoS One. 2023 Dec 27;18(12):e0286441. doi: 10.1371/journal.pone.0286441 (PMC10752528; doi:10.1371/journal.pone.0286441)
Supplement: S1 Fig — All adjusted for age group, sex, BMI, smoking, alcohol, T stage, N stage, tumor size, differentiation, lymphovascular invasion, neural invasion, chemotherapy, and time since cancer diagnosis. (DOC) [file pone.0286441.s006.doc]

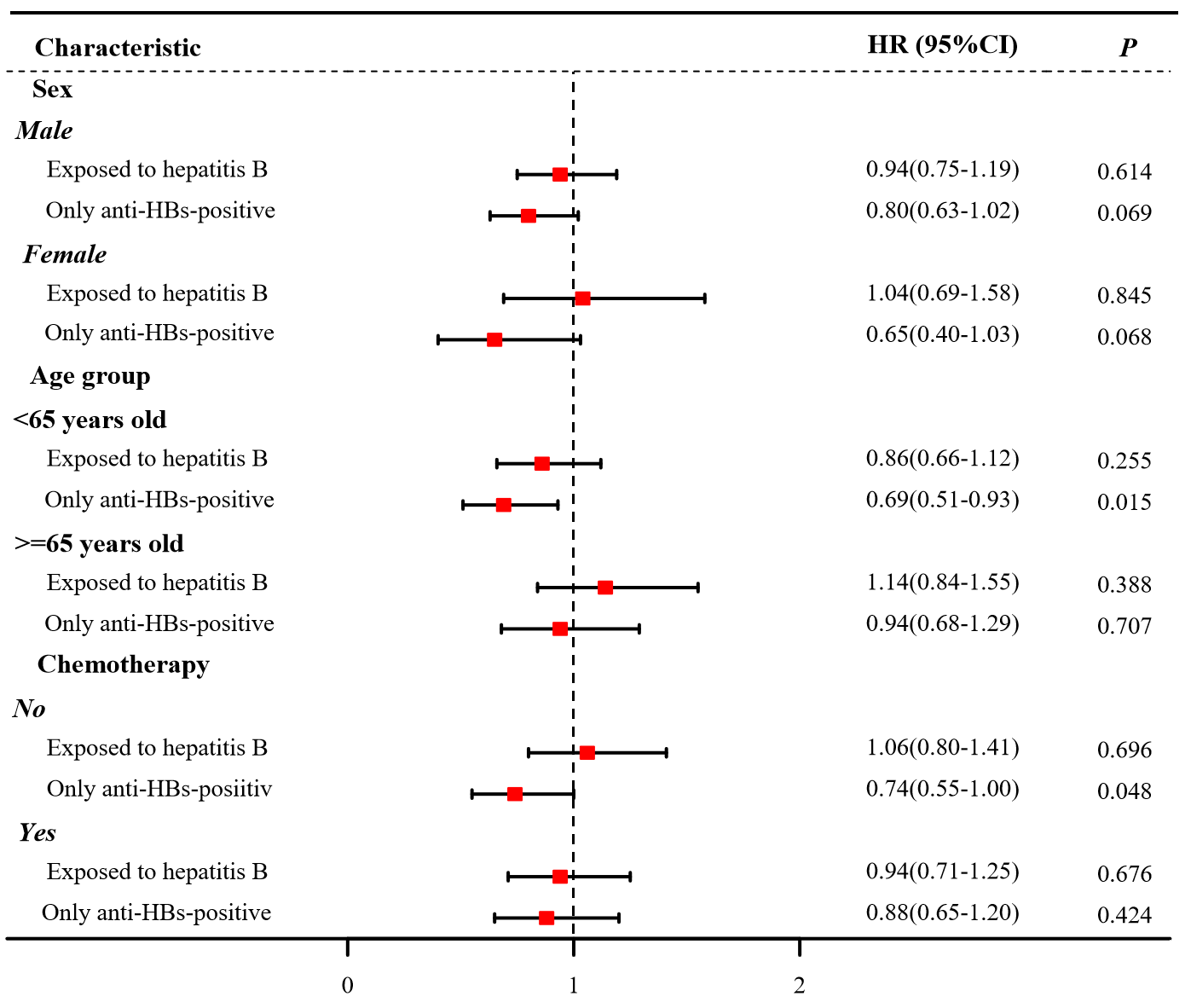


**S1 Fig. Stratified analysis of the association of HBV infection with the risk of all-cause mortality among gastric cohort.**All adjusted for age group, sex, BMI, smoking, alcohol, T stage, N stage, tumor size, differentiation, lymph vascular invasion, neural invasion and chemotherapy and time since diagnosis of cancer.
