# Supplementary material for: Predictive value of hepatitis B serological indicators for mortality among cancer survivors and validation in a gastric cancer cohort
Source: PLoS One. 2023 Dec 27;18(12):e0286441. doi: 10.1371/journal.pone.0286441 (PMC10752528; doi:10.1371/journal.pone.0286441)
Supplement: S2 Fig — A. NHANES; B. Gastric cancer cohort. (DOC) [file pone.0286441.s007.doc]

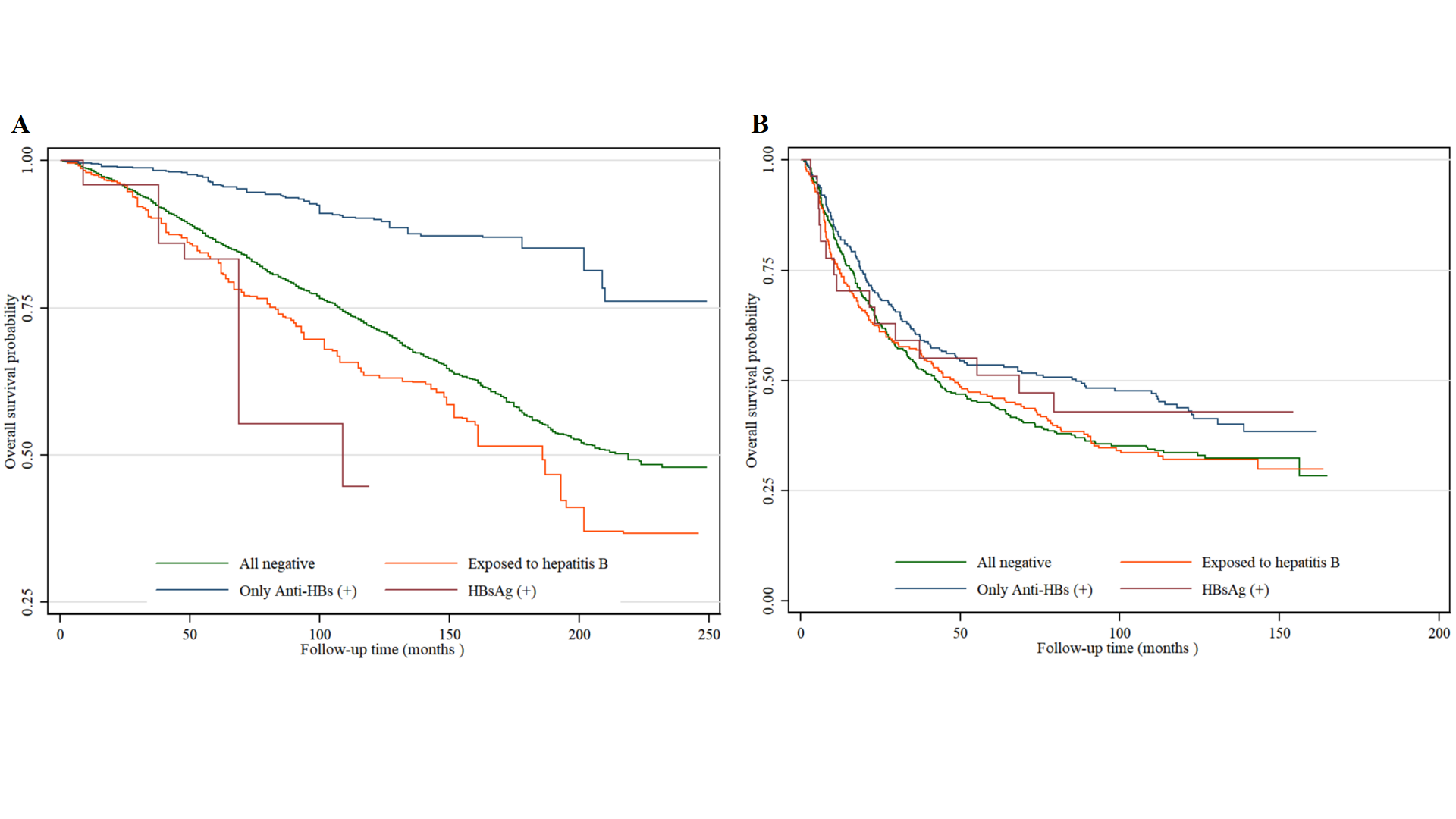


**S2 Fig. Kaplan-Meier estimates of overall survival with different HBV infection status.** A. the NHANES; B. the gastric cancer cohort.
