# Supplementary figures and images for: Predictive value of hepatitis B serological indicators for mortality among cancer survivors and validation in a gastric cancer cohort
Source: PLoS One. 2023 Dec 27;18(12):e0286441. doi: 10.1371/journal.pone.0286441 (PMC10752528; doi:10.1371/journal.pone.0286441)

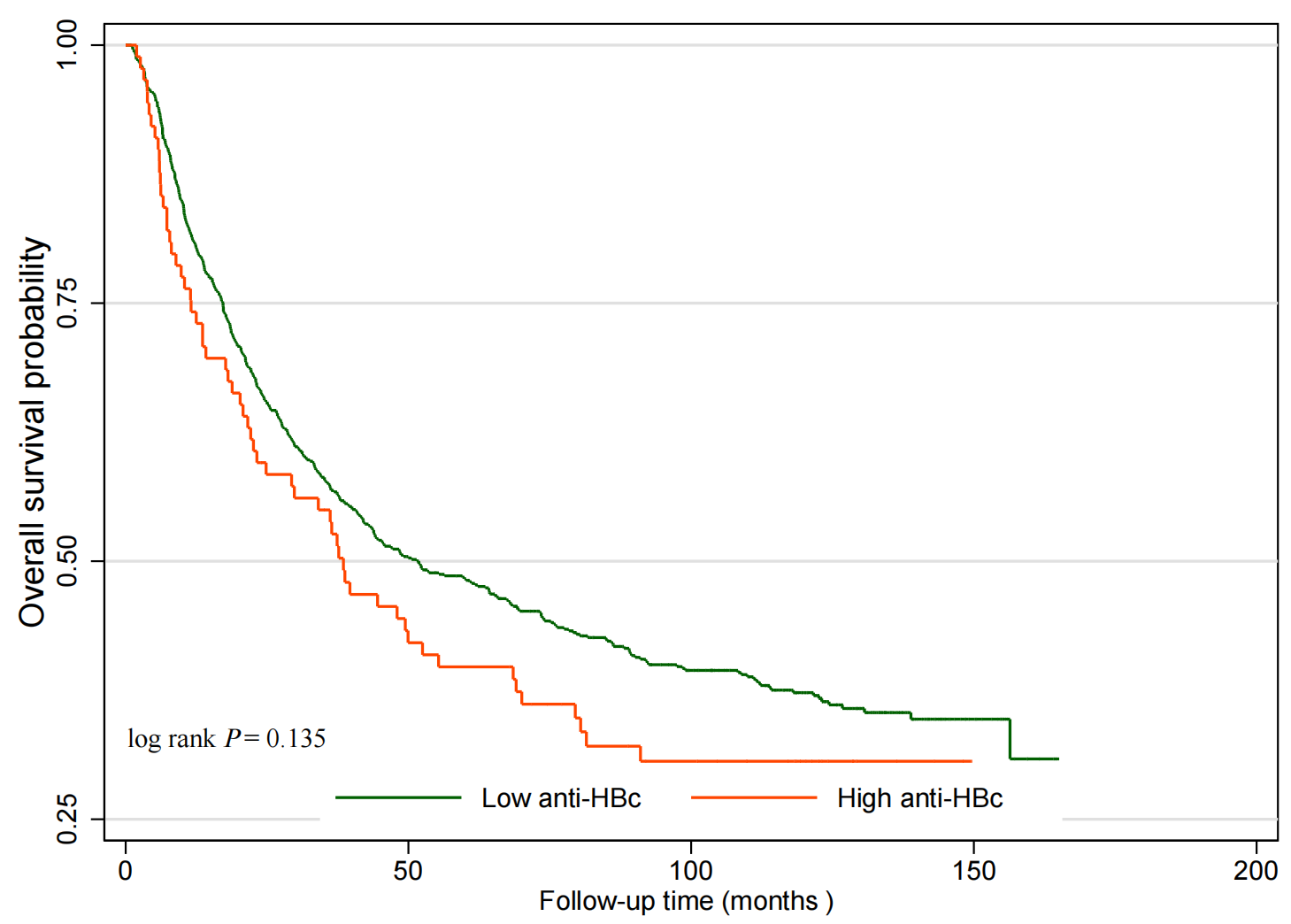


**S3 Fig. Kaplan-Meier estimates of overall survival with different titers of anti-HBc (cutoff: 8.42 S/CO).**

Supplement: S3 Fig — (DOC) [file pone.0286441.s008.doc]
